# ACTH-independent Cushing’s syndrome with bilateral cortisol-secreting adrenal adenomas: a case report and review of literatures

**DOI:** 10.1186/s12902-018-0250-6

**Published:** 2018-04-23

**Authors:** Jia Wei, Sheyu Li, Qilin Liu, Yuchun Zhu, Nianwei Wu, Ying Tang, Qianrui Li, Kaiyun Ren, Qianying Zhang, Yerong Yu, Zhenmei An, Jing Chen, Jianwei Li

**Affiliations:** 1 Department of Endocrinology and Metabolism, West China Hospital, Sichuan University, Chengdu, 610041 China; 2 Department of Urology, West China Hospital, Sichuan University, Chengdu, 610041 China; 3 Department of Pathology, West China Hospital, Sichuan University, Chengdu, 610041 China; 4 Department of Pathology, Immunology, and Laboratory Medicine, University of Florida, Gainesville, FL 32610 USA

**Keywords:** Bilateral cortisol-secreting adrenal adenomas, Cushing’s syndrome, Adrenal venous sampling, Aldosterone

## Abstract

**Background:**

Adrenocorticotropic hormone (ACTH)-independent Cushing’s syndrome (CS) with bilateral cortisol-secreting adenomas has been rarely reported in the literatures. Precise recognition and management of this disorder constitute a challenge to clinicians due to the difficulty of exact location of the functional lesions.

**Case presentation:**

We herein report a new case of a Chinese female patient with a complaint of exertional dyspnea for over 10 years. ACTH-independent CS was diagnosed based on undetectable ACTH and unsuppressed cortisol levels by dexamethasone. Computed tomography (CT) scan indicated bilateral adrenal masses, and adrenal venous sampling (AVS) adjusted by plasma aldosterone revealed hypersecretion of cortisol from both adrenal glands. Bilateral cortisol-secreting adrenal adenomas were suspected and confirmed by the postoperative pathology in subsequent two-step bilateral laparoscopic adrenalectomy. The symptoms and signs of CS relieved after surgery with continuous glucocorticoid replacement.

**Conclusions:**

AVS adjusted by plasma aldosterone could be a useful technique in diagnosing ACTH-independent CS with bilateral adrenal adenomas prior to surgery. And the aldosterone ratio could be used to confirm the success of adrenal vein cannulation in this situation.

## Background

Cushing’s syndrome (CS), which results from prolonged excessive cortisol secretion, is a collection of complicated symptoms and associated with significant morbidity and mortality [1, 2]. Endogenous CS includes ACTH-dependent and ACTH-independent etiologies, the latter accounts for 15~ 20% of the cases and is usually induced by unilateral adrenal adenomas or adrenal carcinomas accompanied by autonomous adrenal cortisol secretion [1]. ACTH-independent CS is occasionally caused by bilateral adrenocortical lesions, including unilateral functional adenoma with a contralateral non-functional mass, bilateral ACTH-independent macronodular adrenal hyperplasia (AIMAH), bilateral primary pigmented nodular adrenocortical disease (PPNAD), and an extremely rare entity, bilateral adrenocortical tumors [3]. Determining the nature and function of bilateral adrenal masses is always a challenge in clinical practice [4, 5]. We herein report a new case of a Chinese female patient with ACTH-independent Cushing’s syndrome due to bilateral cortisol-secreting adenomas, which was diagnosed through adrenal venous sampling (AVS) adjusted by plasma aldosterone and subsequently confirmed by postoperative pathology. In addition, similar cases in the literatures were briefly summarized for discussion.

## Case presentation

A 55-year-old Chinese female was admitted to our hospital complaining of exertional dyspnea for more than 10 years. She had been developing truncal obesity and facial rounding over the past 2 years, without evidence of acne, hirsutism or wide purple striae. The patient had a family history of hypertension and was diagnosed with hypertension 10 years prior to admission, and she had been using irbesartan, metoprolol and nifedipine XR since then. She was also diagnosed with hyperlipidemia and prescribed with statins for 5 years. The patient reported no history of alcohol or drug abuse, in particular, no history of steroid use.

Physical examination on admission showed elevated blood pressure (164/104 mmHg) and normal heart rate (74 beats per minute). The patient’s height, body weight and waist circumference were 156 cm, 51 kg and 88 cm, respectively, with a body mass index (BMI) of 20.96 kg/m^2^. She had a plethoric moon-shaped face, centripetal obesity, buffalo hump, accompanied by ecchymosis and slight edema at both lower limbs. Neurological examination was unremarkable except for slight muscle weakness of the lower-extremities.

Routine laboratory examinations showed normal complete blood cell count and hepatorenal parameters, whereas the level of serum triglyceride was slightly elevated. The fasting plasma glucose level was 7.33 mmol/L, and glycosylated hemoglobin (HbA1c) was 6.6% (Table 1). Endocrinological examinations showed that circadian rhythm of cortisol disappeared, and the level of ACTH was less than 1.00 ng/L (Table 2). Twenty-four-hour urine free cortisol (24 h UFC) elevated to 634.8μg/24 h (reference range: 20.26-127.55μg/24 h). The next morning (8 a.m.) serum cortisol level after an overnight 1 mg dexamethasone suppression test (DMST) was 787.5 nmol/L, indicated lack of normal suppression (Table 2). The diagnosis of ACTH-independent Cushing’s syndrome was therefore established.

**Table 1:** Laboratory characteristics at the first admission and 1 year after bilateral adrenalectomy

	First admission	One year after operation	Reference values
WBC (10^9^/L)	8.44	8.75	3.5- 9.5
Hb (g/L)	127	124	115- 150
Plt (10^9^/L)	195	262	100- 300
Glu (mmol/L)	7.33	4.77	3.9- 5.9
ALT (IU/L)	30	23	< 40
AST (IU/L)	35	27	< 35
Cre (umol/L)	52.0	48.0	37.0- 110.0
BUN (mmol/L)	6.05	4.60	3.13- 8.17
LDL-c (mmol/L)	3.29	1.58	< 4.0
TG (mmol/L)	1.95	1.15	0.29- 1.83
K (mmol/L)	3.63	4.04	3.5- 5.3
Na (mmol/L)	145.5	144.4	137.0- 147.0
HbA1c (%)	6.6	6.0	4.5- 6.1
BNP (pg/ml)	813	168	0- 334
cTnT (ng/L)	18.4	12.1	0- 14
CK-MB (ng/mL)	6.20	5.40	< 2.88
Aldosterone (ng/dL)	17.15	–	9.8- 27.5
PRA (ng/mL.h)	6.62	–	0.93- 6.56
ARR (ng/dL: ng/mL.h)	2.59	–	–
Plasma norepinephrine (ng/L)	152	–	174- 357
Plasma epinephrine (ng/L)	76	–	60- 104
Urinary norepinephrine (μg/24 h)	8.68	–	16.3-41.5
Urinary epinephrine (μg/24 h)	3.21	–	7.5-21.9

*ALT* alanine aminotransferase, *ARR* aldosterone-to-renin ratio, *AST* aspartate transaminase, *BNP* brain natriuretic peptide, *BUN* blood urea nitrogen, *CK-MB* creatine kinase-MB, *Cre* creatinine, *cTnT* cardiac troponin T, *Glu* glucose, *Hb* hemoglobin, *HbA1c* glycosylated hemoglobin, *K* potassium, *LDL-c* low-density lipoprotein cholesterol, *Na* sodium, *Plt* platelet, *PRA* plasma renin activity, *TG* triglyceride, *WBC* white blood cell count

**Table 2:** Results of hormone levels and dexamethasone suppression tests

		08:00 ACTH (ng/L)	24 h UFC (ug/24 h)	24:00 PTC (nmol/L)	08:00 PTC (nmol/L)	08:00 PTC the next day (nmol/L)
Before operation	Baseline	< 1.0	634.8	716.0	858.4	–
	1 mg ODMST	–	–	–	767.0	787.5
After right adrenalectomy	Baseline	< 1.0	40.8	202.1	223.9	–
	1 mg ODMST	–	–	–	211.6	290.1

*ACTH* adrenocorticotropic hormone, *ODMST* overnight dexamethasone suppression test, *PTC* plasma total cortisol, *24 h UFC* 24 h urine free cortisol

For differential diagnosis, aldosterone-to-renin ratio (ARR) was measured after discontinuation of irbesartan and nifedipine XR for at least 2 weeks as they might lead to false-negative result. Plasma and urinary catecholamine concentrations were detected as well. The diagnosis of primary aldosteronism (PA) was excluded since both plasma renin activity (PRA) and aldosterone concentration (PAC) were within normal limits along with an ARR value of 2.59 ng/dL: ng/mL.h. Pheochromocytoma was also ruled out based on laboratory findings (Table 1).

**Fig. 1:**
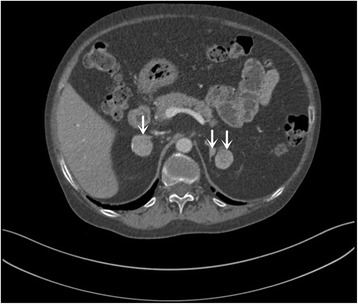
Adrenal computed tomography (CT) of the patient. Adrenal CT showed a right adrenal nodule with a diameter of 2.5 cm, and two left nodules with diameters of 2.3 cm and 0.6 cm, respectively (arrows)

Three adrenal nodules were found with adrenal contrast-enhanced CT. One on the right side was 2.5 cm in diameter, and the other two on the left side with diameters of 2.3 cm and 0.6 cm, respectively (Fig. 1). Magnetic resonance imaging (MRI) of sellar region revealed normal findings. Bone mineral density measured by dual-energy X-ray absorptiometry scans showed that the T score of lumbar spine, femoral neck and the total hip was − 3.0, − 3.2 and − 3.3, respectively, which indicated osteoporosis. In order to locate the functional lesions in this patient, AVS was performed and the concentrations of plasma aldosterone and cortisol were measured from both adrenal veins (AV) and inferior vena cava (IVC). Adrenal venous catheterization was successful, and the hormone levels were shown in Table 3. The adrenal vein to inferior vena cava cortisol (AV: IVC) gradient was 13.57 on the right side and 13.88 on the left side. The left and right AV to IVC gradient of aldosterone were 5.58 and 6.79 respectively. Moreover, the cortisol/aldosterone ratio (CAR) in adrenal veins was 292.52 on the right and 359.29 on the left, along with a left-to-right odds ratio of 1.23 (Table 3). In combination with the results of AVS, which indicated non-lateralization, this patient was diagnosed with CS induced by bilateral adrenal excessive cortisol secretion.

**Table 3:** Results of adrenal venous sampling

	Aldosterone (ng/dL)	Cortisol (nmol/L)	Cortisol/Aldosterone ratio	Lateralization ratio
Left adrenal vein	27.73	9963	359.29	1.23
Right adrenal vein	33.29	9738	292.52	–
Inferior vena cava	4.97	717.6	–	–
AV: IVC ratio (left/right)	5.58/ 6.79	13.88/13.57	–	–

*AV* adrenal vein, *IVC* inferior vena cava

### Treatment and follow-up

**Fig. 2:**
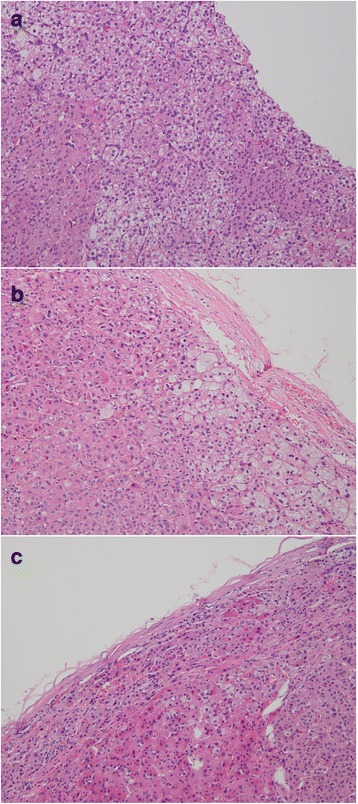
Pathological findings of the resected adrenal glands. Pathological examination of the right (**a**) and left adrenal gland (**b** and **c**) indicated bilateral adrenal adenomas (HE stain, × 200)

The patient was treated with metoprolol succinate, rosuvastatin, insulin, calcium and vitamin D supplements during the investigation. Considering her poor cardiac function, a two-step operation was planned. Laparoscopic right adrenalectomy was performed, followed by left adrenalectomy after a two-month interval. Pathological findings of the removed right adrenal mass indicated a yellow adenoma with 2.5 cm in diameter, surrounded by atrophic adrenal tissue (Fig. 2).

Overnight 1 mg DMST was repeated 2 weeks after surgery, which demonstrated no inhibition on the serum cortisol at 8 a.m. on the following day, despite significantly decreased cortisol level post-operation (Table 2). Therefore, it can be inferred that the autonomous cortisol secretion from left adrenal masses was persistent. The left adrenal gland was then removed and two adenomas were confirmed by pathological examination (Fig. 2). The 8 a.m. plasma cortisol after 3 days of bilateral adrenalectomy was 37.30 nmol/L. Hydrocortisone replacement therapy (from 20 mg t.i.d to 20 mg q.d.) was administered after surgery. At 1 year after the operations, the patient lost 4 kg of body weight and the waist circumference reduced to 71 cm. Changes of other laboratory examinations at the last follow-up compared with the first admission were shown in Table 1.

### Literature review

An electronic literature search in PubMed was performed to screen the case reports relating to ACTH-independent Cushing’s syndrome caused by bilateral cortisol-secreting adenomas. Searching words included “Cushing’s syndrome” and “bilateral adrenocortical adenoma”. All reference lists from the main reports and relevant reviews were screened manually for additional eligible studies. The results were limited to full-text articles published in English. Extracted data included the first author’s name, year of publication, country, preoperative diagnostic technique, patient characteristics (gender, age at onset and at diagnosis), lesions size, operative method and tumor cut surface.

**Fig. 3:**
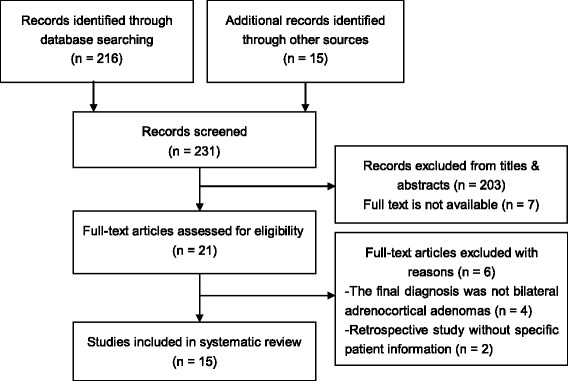
Flowchart of study selection for the literature review

A total of 231 papers were identified, of which, 15 available reports were included in the review (Fig. 3). The clinical features in these patients were summarized as following: 1) this disorder seemed predominated in females (male: female ratio 1: 14), with an adult onset (the mean age was 39.6 ± 8.6 years; ranged from 24 to 53 years); 2) the size of bilateral adrenal adenomas ranged from 1.0 to 5.0 cm in diameter, the majority of which were solitary in both sides (12 out of 15, 80%); 3) most of the bilateral adrenal lesions were found to occur synchronously, except that three cases occurred at different periods [6, 7]; 4) the surrounded adrenal cortex of resected adenomas was atrophic in most cases; 5) although no recurrence was reported postoperatively, long-term outcomes remain unclear with the longest follow-up duration of 123 months [8] (Table 4).

**Table 4:** The available case reports of CS with bilateral adrenal adenomas

Study ID	Country	Age (years)		Gender	Lesions size (cm)		Preoperative diagnostic technique
		Onset	Diag.		right	left	
1963 Chappell, AG.	NM	43	47	Female	3.0, 1.5	3.0	Left AVS and clinical manifestation
1985 Mimou N.	Japan	NM	43	Female	2.0	2.5	AVS without correction and Adrenocortical scintigraphy with ^75^Se-Scintadren
1995 Dinneen SF.	America	49	69	Female	3.1	2.1	AVS without correction
1997 Makino, S.	Japan	35	45	Female	2.5, 2.0	2.4	Adrenocortical scintigraphy with ^131^I-adosterol
1997 Tamura, H.	Japan	43	48	Female	3.5	2.4	Adrenocortical scintigraphy with ^131^I-adosterol
2003 Nomura, K.	Japan	33	37	Female	1.8	1.6	Adrenocortical scintigraphy with ^131^I-NP-59
2004 Tung SC.	Taiwan	24	33	Female	3.0	3.0	Adrenocortical scintigraphy with ^131^I-NP-59
2006 Desai, N.	America	30	30	Female	2.0	3.0	Iodocholesterol scan
2006 Inoue T.	Japan	39	41	Female	2.0	2.4	Adrenocortical scintigraphy
2007 Domino, JP.	Singapore	NM	35	Female	1.8	2.2	AVS adjusted by aldosterone
2008 Oki, Kl.	Japan	35	50	Female	1.1	1.0	AVS adjusted by aldosterone and Adrenocortical scintigraphy with ^131^I-adosterol
2012 Martins, RG.	UK	51	59	Female	2.8	2.6	AVS without correction
2013 Ku, EJ.	Korea	45	48	Female	2.8	2.3, 1.7	AVS without correction
2014 Yasuda, A.	Japan	53	63	Male	2.4	2.5	AVS without correction and Adrenocortical scintigraphy with ^131^I-adosterol
2015 Monno, S.	Japan	35	39	Female	2.4	3.1	Adrenocortical scintigraphy with ^131^I-NP-59
Study ID	Operation		Cut surface				Functional side
			right		left		
1963 Chappell, AG.	Left, right total adrenalectomy (2 months later)		Yellow		NM		NM
1985 Mimou N.	Bilateral total adrenalectomy		Dark brown		Brownish-yellow		Bilateral adenomas
1995 Dinneen SF.	Bilateral total adrenalectomy		NM		NM		Bilateral adenomas
1997 Makino, S.	Bilateral total adrenalectomy		Yellow, Black		Yellow		Left adenoma, the black right adenoma
1997 Tamura, H.	Bilateral total adrenalectomy		Brown		NM		Bilateral adenomas
2003 Nomura, K.	Bilateral subtotal adrenalectomy		Black		Black		Bilateral adenomas
2004 Tung SC.	Right, left total adrenalectomy (9 years later)		Yellow		Yellow		Bilateral adenomas
2006 Desai, N.	Bilateral total adrenalectomy		Yellow/ tan		Yellow/ tan		Bilateral adenomas
2006 Inoue T.	Bilateral partial adrenalectomy		Yellowish-brown		Yellowish-brown		Bilateral adenomas
2007 Domino, JP.	Right total adrenalectomy, left partial adrenalectomy		NM		NM		Bilateral adenomas
2008 Oki, Kl.	Left partial adrenalectomy, right total adrenalectomy		Golden yellow		NM		Bilateral adenomas
2012 Martins, RG.	Bilateral total adrenalectomy		NM		NM		Bilateral adenomas
2013 Ku, EJ.	Bilateral total adrenalectomy		Brown		Light brown		Bilateral adenomas
2014 Yasuda, A.	Left total adrenalectomy, right partial adrenalectomy		NM		NM		Bilateral adenomas
2015 Monno, S.	Left, right total adrenalectomy (3 months later)		Yellow		Golden		Bilateral adenomas

*AVS* adrenal venous sampling, *CS* Cushing’s syndrome, *NM* not mentioned, ^*75*^*Se-Scintadren* 6-methyl-^75^Se-selenomethyl-19-norcholeste-5(10)-en-3ß-ol, ^*131*^*I-NP-59* ^131^I-6β-iodomethyl-19-norcholesterol

All preoperative diagnoses were established based on endocrinological studies and imaging findings, while the methods used to determine the functional lesions were different. Nine patients underwent adrenocortical scintigraphy with different radio-imaging agents, all of which revealed bilateral adrenal uptake. AVS was performed in eight cases to evaluate the hypersecretion of cortisol, and only two of them applied cortisol gradient adjusted by plasma aldosterone [9, 10]. All patients underwent surgical resection of adenomas, including ten bilateral total adrenalectomy [6, 11–19], three unilateral partial adrenalectomy with contralateral total adrenalectomy [9, 10, 20], one bilateral partial adrenalectomy [21] and one bilateral subtotal adrenalectomy [22]. All patients received glucocorticoid replacement therapy postoperatively. It is noteworthy that glucocorticoid therapy was reported to be withdrawn during follow-up in patients who underwent bilateral subtotal adrenalectomy or partial adrenalectomy [21–23].

## Discussion and conclusion

ACTH-independent Cushing’s syndrome with bilateral cortisol-secreting adenomas has been rarely reported in the literature [6, 8–24]. This disorder should be differentiated from PPNAD, AIMAH and unilateral functional adenoma with contralateral non-functional lesion for the determination of therapeutic regimen. PPNAD is characterized by multiple small pigmented nodules of hyperplastic adrenocortical cells and cortical atrophy with an early age of onset [25]. AIMAH, in which the bilateral enlarged adrenal glands with numerous nodules larger than 1 cm in diameter lead to an irregular contour on CT or MRI, is associated with aberrant expression of hormone receptors and can be treated by appropriate antagonist [26, 27]. The definite diagnosis of AIMAH or PPNAD depends on histo-pathology. Although the clinical characters of some similar cases were identified by several previous studies, precise diagnosis and treatment of patients with bilateral ACTH-independent adrenal adenomas remain challenging [8, 23, 24].

The diagnostic value of AVS and ^131^I-6β-iodomethyl-19-norcholesterol (^131^I-NP-59) scintigraphy for defining the hormone-secreting status in adrenocortical diseases was well established [28–30]. NP-59, which was clinically available in 1975 and most frequently used, can be accumulated by functioning adrenal cortical tissues as radiolabeled cholesterol analog [31]. The uptake of NP-59 would reflect the anatomic localization and functional characterization of adrenal masses accordingly. Since the ^131^I-NP-59 scintigraphy is unavailable in most hospitals in China and many other countries, an alternative technique is needed for the diagnosis.

AVS has been recommended over the past 10 years by international guidelines to differentiate unilateral from bilateral primary aldosteronism through cortisol-corrected aldosterone ratio [30, 32, 33]. During AVS, blood was collected from bilateral AV and IVC or peripheral vein (PV) to measure aldosterone and cortisol levels, and the comparison of AV and IVC cortisol concentration was further used to assess whether successful cannulation was achieved in PA. In this case, we used the AV: IVC aldosterone ratio to assess the successfulness of catheter insertion since the aldosterone concentration could remain stable during the sampling. There were some researches in which adrenaline concentration was measured to evaluate catheterization accuracy, however, catheterization process itself might lead to stress-induced fluctuation of adrenaline and further result in misjudgment [17, 34]. Even though there is still no general agreement on the definition of catheterization success in AVS, aldosterone concentration instead of adrenaline could be a robust assay for this purpose, after the aldosterone overproduction being excluded.

We used AV: IVC cortisol ratio in both side and left-to-right CAR gradient to differentiate unilateral from bilateral cortisol overproduction. Young et al. [8] suggested that if the cortisol gradient of AV to PV or IVC was greater than 6.5, cortisol-secreting adenoma should be considered. Although the accuracy and applicability remain to be proved due to the lack of research in this area, we can at least assume that a larger ratio represents a greater likelihood of spontaneous cortisol secretion. The authors also proposed cutoff values of high- to low-side AV cortisol gradient ratio to determine the lateralization of cortisol hypersecretion, and suggested that predominant cortisol secretion was considered if the cortisol lateralization ratio was ≥2.3 [8]. However, unadjusted cortisol lateralization ratio might confound the interpretation of AVS results because of the cortisol concentration was 1.8 times higher in the right AV than in the left side, which was the result of dilution effect [35]. Since PA was ruled out in this patient, the correction was considered to be achieved through aldosterone in this setting. It should be noted, however, that AVS cannot be used to differentiate the functional status of each mass in the left adrenal gland.

AVS is generally safe, with a very low risk of adverse events. The rate of adrenal venous rupture, one of the main complications, was reported as 0.61% in a recent international multicenter study [36]. Adrenal vein thrombosis, infarction and perforation, and subsequent periadrenal hemorrhage and hematoma have been also reported in the literature [37]. In consideration of the risk of failure and complications, experienced radiologists were suggested for this invasive procedure. In this case, no adverse event was observed throughout the duration of treatment and follow-up.

The optimal treatment for patients with bilateral cortisol-secreting adenomas remains uncertain [27, 38]. Two-step bilateral adrenalectomy was performed on our patient and resulted in remarkable remission of hypercortisolism, and further confirmed the cortisol-secreting feature of each adrenal mass. Even though lifelong steroid supplementation was required, the quality of her life improved considerably. However, lifelong follow-up of the patient is required regarding to the unclear long-term outcome of bilateral adrenalectomy in this disease [39, 40]. Recently, partial adrenalectomy (removal of the adenomas only) was performed in some similar cases, in which functional recovery of hypothalamic-pituitary-adrenal axis could be achieved after surgery, while further studies for prognosis are still warranted [9, 20, 21, 23].

In summary, we reported a Chinese female patient with ACTH-independent CS caused by bilateral cortisol-secreting adenomas. She was diagnosed through aldosterone-adjusted AVS and successfully treated with bilateral adrenalectomy. It confirmed that selective AVS with aldosterone-corrected cortisol ratio could be a useful technique to evaluate the cortisol-secreting function of each adrenal mass and further guide therapeutic decision-making. Owing to the existing dispute over the interpretation of the AVS results, the definite cut-off values for lateralization of cortisol hypersecretion requires further confirmation.

## Abbreviations

^131^I-NP-59: ^131^I-6β-iodomethyl-19-norcholesterol
24 h UFC: Twenty-four-hour urine free cortisol
ACTH: Adrenocorticotropic hormone
AIMAH: ACTH-independent macronodular adrenal hyperplasia
ARR: Aldosterone-to-renin ratio
AV: Adrenal vein
AVS: Adrenal venous sampling
BMI: Body mass index
BNP: Brain natriuretic peptide
CAR: Cortisol to aldosterone ratio
CS: Cushing’s syndrome
CT: Computed tomography
DMST: Dexamethasone suppression test
HbA1c: Glycosylated hemoglobin
IVC: Inferior vena cava
MRI: Magnetic resonance imaging
PA: Primary aldosteronism
PAC: Plasma aldosterone concentration
PPNAD: Primary pigmented nodular adrenocortical disease
PRA: Plasma renin activity
PV: Peripheral vein
